# Pro-Angiogenic Effects of Natural Antioxidants Extracted from Mango Leaf, Olive Leaf and Red Grape Pomace over Endothelial Colony-Forming Cells

**DOI:** 10.3390/antiox11050851

**Published:** 2022-04-27

**Authors:** Ismael Sánchez-Gomar, Josefa Benítez-Camacho, Cristina Cejudo-Bastante, Lourdes Casas, Rafael Moreno-Luna, Casimiro Mantell, Mª Carmen Durán-Ruiz

**Affiliations:** 1 Biomedicine, Biotechnology and Public Health Department, University of Cadiz, 11002 Cadiz, Spain; ismael.sanchez@gm.uca.es (I.S.-G.); josefa.benitezcamacho@alum.uca.es (J.B.-C.); 2 Institute of Research and Innovation in Biomedical Sciences of Cadiz (INIBICA), 11009 Cadiz, Spain; 3 Chemical Engineering and Food Technology Department, Science Faculty, Wine and Agrifood Research Institute (IVAGRO), University of Cadiz, 11519 Cadiz, Spain; cristina.cejudo@gm.uca.es (C.C.-B.); casimiro.mantell@uca.es (C.M.); 4 Laboratory of Neuroinflammation, National Paraplegics Hospital, SESCAM, 45071 Toledo, Spain; rmluna@sescam.jccm.es

**Keywords:** ECFCs, mango leaves, olive leaves, grape pomace, angiogenesis, proliferation, inflammation, cell therapy, polyphenols, antioxidants

## Abstract

Cardiovascular diseases remain the leading cause of death worldwide, mainly triggered by the formation of atherosclerotic plaques that reduce blood flow. Angiogenic cell therapy based on endothelial colony forming cells (ECFCs) constitutes a promising alternative to promote vascular revascularization; however, under the oxidative environment that prevails in ischemic areas, these cells become impaired. Thus, it is necessary to investigate strategies to enhance their regenerative properties. Antioxidant substances, such as polyphenols, have been shown to be useful for this purpose. In the current study we evaluated the potential of mango leaves, olive leaves and red grape pomace extracts, rich in polyphenols, to promote ECFC reparative effects. For this, aqueous and ethanolic extracts of the aforementioned raw materials were obtained by pressurized liquid extraction (PLE). After evaluating the polyphenol content and the antioxidant activity, in vitro assays were carried out, and we found that ethanolic extracts at low concentrations improved angiogenic capacities of ECFCs and reduced proliferation, apoptosis, and the inflammatory response of these cells. Overall, mango leaves ethanolic extract provided the most promising results, but all three extracts ameliorated the functionality of ECFCs.

## 1. Introduction

Cardiovascular diseases (CVDs) are responsible for a high number of annual deaths worldwide, with atherosclerosis the main triggering factor [1]. Atherosclerosis is characterized by a chronic inflammatory response due to the accumulation of fat in the innermost layer of the arteries, the intima, causing the appearance of atherosclerotic plaques, limiting blood flow, and promoting ischemic events. Indeed, depending on the location and severity of the plaque, myocardial infarctions, strokes, or peripheral ischemia may occur [2]. Cell therapy with endothelial colony-forming cells (ECFCs) provides hope in the revascularization of ischemic areas in atherosclerotic patients [2,3]; however, these cells become dysfunctional in the presence of risk factors that predispose a patient to the onset of ischemic disorders [4]. In this sense, current strategies seeking to enhance their regenerative potential even under pathological conditions are being designed [4]. For example, the incubation with human platelet lysate boosted ECFCs expansion [5], while preconditioning with fucoidan [6], erythroid growth factor EPO [7], or nicotine [8] improved ECFCs proliferation. This proliferation is also stimulated by causing an increase in the intracellular Ca^2+^ [9].

Similarly, the capacity of some polyphenols to ameliorate therapeutic angiogenesis of endothelial progenitor cells (EPCs) has also been evaluated. Polyphenols are secondary plant metabolites known for their strong antioxidative properties. They are abundant in many healthy dietary patterns and play an important role in the prevention of diseases associated with oxidative stress and inflammation [10]. In CVDs, polyphenols reduce endothelial dysfunction by inhibiting low density lipoprotein (LDL) oxidation, reducing reactive oxygen species (ROS) generation, decreasing the production of proinflammatory cytokines and preventing platelet aggregation and the formation of atherosclerotic plaques [11,12]. Notably, ELN/41 or Proxison, a novel synthetic flavonoid, was able to improve ECFCs angiogenic expansion when introduced into explants of ischemic choroids from P8 C57BL/6J mice [13]. Furthermore, the flavonoids quercetin, kaempferol, and myricetin seem capable of reducing oxidative stress and apoptosis on EPCs in vitro [14]. Quercetin was reported to enhance the viability and migration of EPCs at a dose-dependent manner by activating the phosphorylation of protein kinase B or AKT, endothelial nitric oxide synthase (eNOS) and the protein extracellular signal-regulated kinase (ERK) [15]. Alternatively, it was found that genistein promoted proliferation and migration of ECFCs in vitro, and the transplantation of ECFCs pre-stimulated with this polyphenol into myocardial ischemic sites promoted neovascularization and improved cardiac function in vivo [16].

The application of high-pressure-based extraction techniques such as pressurized liquid extraction (PLE) has significantly benefited this field, since phenolic extracts are currently easier to obtain, minimizing solvent consumption and reducing the cost of purification steps [17,18,19]. Indeed, thanks to these approaches, many studies have focused on evaluating the use of mango leaves, olive leaves and red grape pomace extracts, rich in polyphenols, in the treatment of CVDs [20,21,22]. Nevertheless, regarding cell therapy, there is not much information on the use of these extracts on the angiogenic capacities of ECFCs. Thus, bearing in mind that antioxidant and anti-inflammatory properties of extracts rich in polyphenols correlate with an improvement in reparative capacities of endothelial cells (ECs), in the current study we evaluated whether mango leaves, olive leaves, and red grape pomace extracts can improve angiogenic capacities of ECFCs, along with their effect on proliferation, differentiation towards more mature ECs, apoptosis, and inflammation.

## 2. Materials and Methods

### 2.1. Raw Material

The raw materials used to obtain the extracts were mango leaves, provided by the Institute for Mediterranean and Subtropical Horticulture ‘La Mayora’ (IHSM) of the Spanish National Research Council (CSIC; Malaga, Spain), olive leaves (San José de Lora de Estepa olivarera Sca Coop, Seville, Spain), and red grape pomace (Luis Pérez wineries, Jerez, Spain). Mango and olive leaves were collected in 2018 and 2019, respectively. All raw materials were dried under ambient conditions and stored at room temperature in the absence of light.

### 2.2. Chemicals and Reagents

Ultrapure water (Milli-Q) and ethanol (96%) (EtOH) were obtained from Panreac (Barcelona, Spain). 2,2-Diphenyl-1-picrylhydrazyl (DPPH), Folin-Ciocalteu reagent, sodium carbonate, gallic acid monohydrate, potassium chlorate, sodium acetate, and hydrochloric acid were supplied by Sigma–Aldrich (Steinheim, Germany).

### 2.3. Pressurized Liquid Extraction (PLE)

Mango leaves (*Mangifera indica* L. cv Kent), olive leaves (*Olea europaea*) and red grape pomace (*Vitis vinifera*) extracts were obtained by PLE. Extractions were carried out in high pressure extractors (SF1000 and SF2000, Thar Technology), fitted with a thermostatted vessel with a capacity of 1–2 L, two double piston high pressure pumps (Thar Technology model P100 for carbon dioxide and P50 for the co-solvent), a preheater, a back pressure regulator valve (BPR) and a cyclonic separator. In order to compare the yields and antioxidant capacity of the extracts obtained, two solvents were analyzed in the present work, ultrapure water (Milli-Q) and ethanol (96%), both provided by Panreac (Barcelona, Spain).

The working conditions were set at a pressure of 200 bar, a temperature of 80 °C and an extraction time of 12 h in static mode, based on previous studies [17,23]. A sample quantity between 100 and 500 g was introduced depending on the type of extractor used.

### 2.4. Extraction Yield and Total Polyphenol Content (TPC)

The concentration and the extraction yield were determined by gravimetry. The total polyphenol content (TPC) was obtained by a test based on the Folin–Ciocalteu method [24], analysing equivalent gallic acid concentration (GAEq) in the extracts.

### 2.5. Antioxidant Activity by DPPH Method

The antioxidant activity of the extracts was determined by the method previously described [25,26], based on the property of the 2,2-diphenyl-1-picrylhydracil (DPPH) free radical to absorb at a wavelength of 515 nm, and in the loss of its absorption capacity when it is reduced by an antioxidant substance [27].

### 2.6. Selection of Extracts

Based on their antioxidant activity, several concentration ranges were tested in a viability test, to determine in which concentrations cell viability remained (Supplementary Figure S1). Thus, two concentrations per extract were selected: the lowest at which an improvement in the viability of the culture was observed, and the highest at which the culture was kept viable: Mango-H_2_O (11.67 and 186.67 μg/mL); Mango-EtOH (4.92 and 26.25 μg/mL); Olive-H_2_O (140 and 560 μg/mL); Olive-EtOH (46.67 and 140 μg/mL); Grape-H_2_O (466.67 and 1866.67 μg/mL); Grape-EtOH (11.67–93.34 μg/mL).

Next, an angiogenesis test was performed, to obtain a first approximation of how these extracts affect the angiogenic capacity of ECFCs and to select only the optimal concentration at which these extracts promote angiogenesis to a greater extent (Supplementary Figure S2).

Based on these preliminary results, further experiments were carried out with the lowest concentrations of the ethanolic extracts: Mango-EtOH (4.92 μg/mL), Olive-EtOH (46.67 μg/mL), grape-EtOH (11.67 μg/mL).

### 2.7. ECFCs Culture and Cell Characterization

Endothelial colony forming cells (ECFCs) were isolated from white adipose tissue, as described [28]. Briefly, ECFCs were cultured in 75 cm^2^ flasks, treated with 1% gelatin (G1890, Sigma, Steinheim, Germany) and incubated in 20% FBS/ EBM-2 media (CC-3156, Lonza, Basel, Switzerland) containing 1% penicillin/streptomycin (P/S) and the necessary growth factors (VEGF, hFGF-B, hEGF and R3-IGF-1), ascorbic acid and heparin, without hydrocortisone (CC-4147, Lonza). Cells were grown until confluence (90%) was reached. Experiments were performed on passages 6–7.

Cell identity was confirmed by testing cloning-forming ability, as described [2], and also by flow cytometry, analyzing several specific antibodies against CD31, CD14, CD90, CD34, CD45, CD73, CD133, CD309 and CD146 (Supplementary Figure S3). An isotype IgG1 antibody was used for negative control. The full list of antibodies is shown in Supplementary Table S1. Fluorescence was measured using CytoFLEX cytometer (Beckam Coulter, West Sacramento, CA, USA) and CytExpert software. Finally, data were analyzed with FlowJo v10.4 software.

### 2.8. Angiogenesis Assay

The effect of the extracts on the angiogenic capacity of ECFCs was determined by a tube formation assay test, using a matrigel support, as described [29]. Briefly, 15,000 cells were seeded in μ-plate angiogenesis 96 well (ibidi, Fitchburg, WI, USA, 89,646) pre-coated with 10 μL Matrigel (BD Bioscience, San Jose, CA, USA, 256,231) as described [2], and incubated with the ethanolic extracts at the preselected concentrations: mango (4.92 μg/mL), olive (46.67 μg/mL), grape (11.67 μg/mL), in a total volume of 50 µL/well of EBM-2, 5% FBS, 1% P/S. In addition, a negative control, with ECFCs in basal medium (EBM-2. 5% FBS, 1% P/S) was included, as well as an angiogenesis inhibition control with ECFCs and 15 mM sulforaphane (S4441-5MG, Sigma) and an angiogenesis activation control, incubating ECFCs with Fibroblast Growth Factor (FGF) at 35 ng/mL (R&D Systems). All conditions were evaluated in triplicate. Cells were incubated at 37 °C, 5% CO_2_ in a humid chamber. Photographs were taken after 24, 48 and 72 h with the 4X objective (AE2000 Series, Motic). Images were analyzed with the Angiogenesis Analyzer plugin of ImageJ software, measuring the number of meshes, the number of segments, and the total length.

### 2.9. Proliferation-Differentiation Assay

ECFCs were seeded (35,000 cells/well) in 24-well plates containing round coverslips (1.3 cm diameter) pre-treated with 1% gelatin, incubated with 500 µL of basal medium for 24 h at 37 °C, 5% CO_2_, and then incubated with the extracts at the preselected concentrations, in triplicates.

After 48 h, cells were washed with PBS 1x, fixed with 4% paraformaldehyde and permeabilized with 0.2% triton in 1x PBS (0.2% PBT) for 30 min in agitation. Blocking was made 2.5% bovine serum albumin (BSA), in 0.2% PBT, prior incubation at 4 °C overnight, with the rabbit anti-Ki67 primary antibody (PA5-16785; Invitrogen, Waltham, MA, USA, EEUU) diluted 1:500, and the mouse anti-human vWF antibody (MA5-14029, Thermo Fisher Scientific, Waltham, MA, USA, EEUU) diluted 1:200 in 2.5% BSA. Next, secondary antibodies anti-rabbit 488 (A-11008; Thermo Fisher Scientific) (1:1000) and goat anti-mouse 555 (A-21422: Thermo Fisher Scientific, EEUU) (1:500) were used, 1 h in the dark at RT, followed by incubation with 4’,6-diamidino-2-phenylindole (DAPI) (1:5000 in 2.5% BSA) for nuclei staining. Coverslips were mounted on slides using mounting solution (S302380-2; Dako-Agilent Technologies, Santa Clara, CA, USA, EEUU). Photographs were taken for each replica at 10X, with an Olympus IX81 fluorescence microscope, and images were subsequently analyzed with ImageJ software. DAPI nuclear labelling allowed total cells to be counted, Ki67 positive staining identified proliferating cells, and von Willebrand factor (vWF) positive labelling marked differentiated cells. The proliferation and differentiation percentage of each experimental condition was determined.

### 2.10. Apoptosis Assay

Apoptosis was evaluated by flow cytometry using V450 Annexin V (AN-V) (560,506, BD Biosciences) and propidium iodide (IP) (556,463, BD Bioscences). ECFCs (7 × 10^4^ cells/well) were seeded in 24-well plates and incubated for 24 h as indicated above, and then treated with the same preselected concentrations of mango, olive and grape, for 24 and 48 h, at 37 °C and 5% CO_2_. A control without extracts was used, in addition to the apoptosis negative controls (without AN-V and/or IP). After incubation, cells were trypsinized, washed twice with PBS and centrifuged. Next, cell pellets were resuspended in 100 μL of 1X AN-V Binding Buffer (556,454; BD Biosciences), and incubated with 4 μL of AN-V and 4 μL of IP for 30 min at 4 °C in the dark. Apoptotic cells were analyzed using CytoFLEX cytometer (Beckam Coulter, USA) and CytExpert software. Finally, data were analyzed with FlowJo v10.4 software.

### 2.11. Anti-Inflammatory Assay

ECFCs (7 × 10^5^ cells/well) were seeded in 24-well plates and incubated with the extracts, as described above, and also with tumor necrosis factor alpha (TNF-α) (0.05 mg/mL). A negative control without extracts and TNF-α was used, as well as a positive control with TNF-α without extracts, in addition to the controls for calibration.

After 4 h of incubation, cells were detached and washed with cytometry buffer (1X PBS, 2.5% FBS, 2 mM EDTA). Then, 4 µL of each antibody was added: APC anti-human CD106 or anti-human VCAM-1 (305,809; Biolegend, San Diego, CA, USA) and PE anti-human E-selectin (322,606; Biolegend). Incubation was carried out for 20 min at 4 °C in the dark. Samples were analyzed using CytoFLEX cytometer (Beckam Coulter, USA) and CytExpert software. Finally, data were analyzed with FlowJo v10.4 software.

### 2.12. Statistical Analysis

Data representation and analysis was performed using GraphPad Prism 9 software and IBM SPSS statistics 25. Data were verified for normal distribution using the Shapiro–Wilk test. For data that followed a normal distribution, the homogeneity of variances was confirmed by the Levene test; differences were calculated using variance analysis ANOVA for multivariate analysis, followed by Tukey’s test post-hoc analysis. For non-parametric data, differences between the groups were calculated with Kruskal–Wallis test and Mann–Whitney U test as post-hoc analysis. Data were represented by box and whisker diagrams, including the median, minimum and maximum values, as well as individual data points. Differences were statistically significant with *p*-values < 0.05.

## 3. Results

### 3.1. Chemical and Functional Characterization of Extracts Obtained by Pressurized Liquid Extraction (PLE)

The global yield and the total phenolic content (TPC) of the extracts obtained by PLE (mango leaves, olive leaves, and red grape pomace extracts), as well as their antioxidant capacity, was measured by the DPPH essay [25,26], represented in terms of efficient concentration (EC_50_) and antioxidant activity index (AAI) [26] respectively.

**Figure 1:**
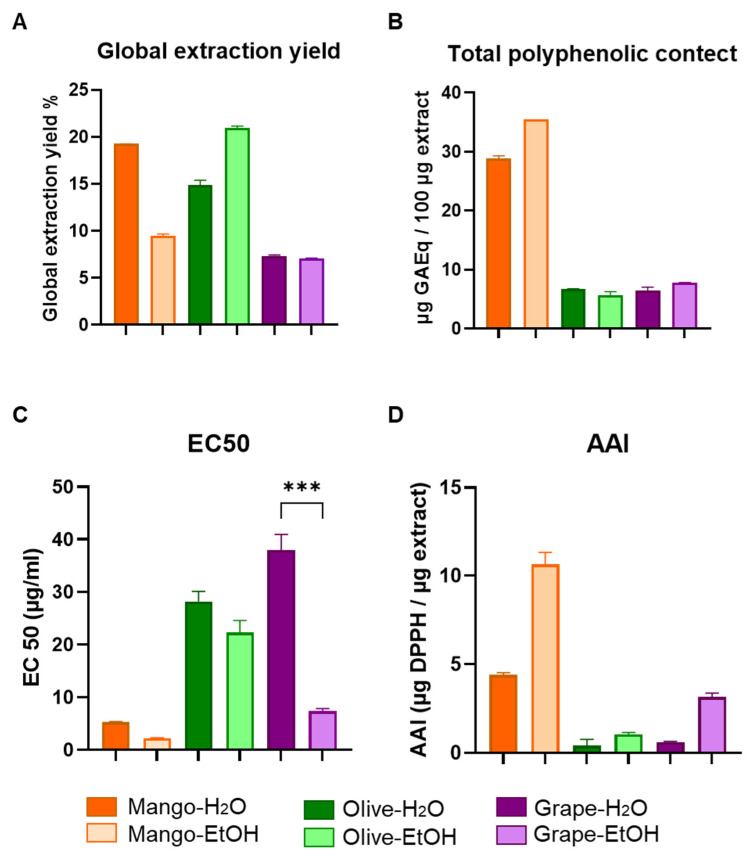
Chemical and functional characterization of the extracts obtained by PLE. (**A**) Graphical representation of global yield of the aqueous and ethanolic extracts of mango leaf, olive leaf, and red grape pomace expressed as g/100 g dry extract (%). (**B**) Total phenolic content expressed as g/100 g dry extract. (**C**) Antioxidant activity represented by the efficient concentration (EC_50_) (µg/mL). (**D**) Antioxidant activity index (AAI) (µg DPPH/µg extract)**.** Values are represented as the mean ± SD. *** *p*-value < 0.001.

Results confirmed that, in the case of mango leaves and grape pomace extracts, a higher yield was obtained in the aqueous extracts than in the ethanolic ones, in contrast to the olive leaves whose higher yield was seen in the ethanolic extract (Figure 1A). TPC was similar in aqueous and ethanolic olive leaves extracts; however, a greater TPC was obtained when using ethanol in both mango leaves and grape pomace extracts (Figure 1B). Overall, the use of pure ethanol led to a more selective extraction of phenolic compounds than water [30]. Finally, in general terms, a higher antioxidant activity was obtained in the ethanolic extracts than in the aqueous ones (Figure 1C,D). Mango leaves ethanolic extracts presented the highest antioxidant activity, followed by red grape pomace and olive leaves (Figure 1C,D). Full information regarding the characteristics of the extracts (polyphenolic and anthocyanin content, antioxidant activity) as well as the extraction techniques can be found in previous works [17,19,31,32].

### 3.2. Selection of the Extracts’ Working Concentrations

Preliminary tests (Supplementary Figures S1 and S2) suggested that ethanolic extracts potentiate the angiogenic properties of ECFCs to a greater extent than aqueous ones, especially when using the following ethanolic extracts concentrations: mango (4.92 μg/mL), olive (46.67 μg/mL), grape (11.67 μg/mL). Furthermore, the incubation with higher concentrations of these extracts reduced the angiogenic capacity. Therefore, we decided to apply these as the experimental concentrations in our study.

### 3.3. Extracts Enhance the Angiogenic Capacity of ECFCs

**Figure 2:**
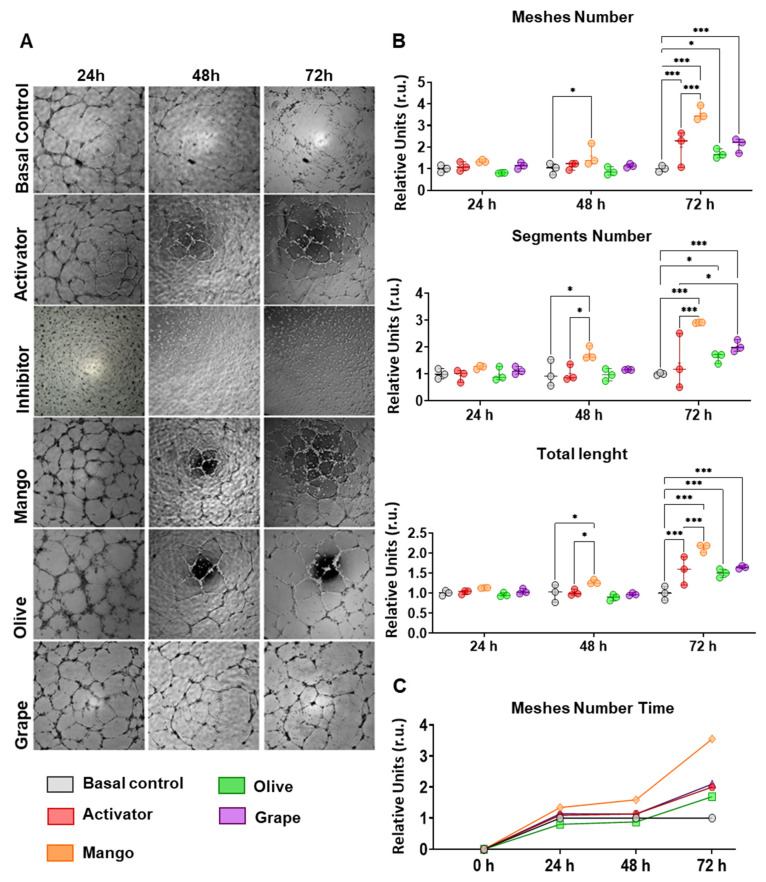
Effect of selected extracts on the angiogenic process of ECFCs. (**A**) Representative images of the reticular structures formed by ECFCs after 24, 48 and 72 h incubation in basal medium, with the inhibitor sulforaphane (15 mM), the activator FGF (35 ng/mL), or with mango (4.99 µg/mL), olive (46.67 µg/mL), and grape (16.67 µg/mL) ethanolic extracts. (**B**) Graphical representation of the changes seen for the number of meshes, number of segments, and total length of the segments analyzed in ECFCs treated with mango leaves, olive leaves, and red grape pomace ethanolic extracts after 24, 48, and 72 h. (**C**) Temporal evolution of the number of meshes in ECFCs treated with the selected extracts. * *p* < 0.05; *** *p* < 0.001.

The incubation of ECFCs with the mango, olive or grape pomace ethanolic extracts did not suggest any significant differences after 24 h incubation with any of the extracts selected, compared to control ECFCs (with basal culture media) or even after incubation with FGF, angiogenic activator (Figure 2). After 48 h, an increase in the number of all parameters (number of meshes, segments, and segment length) was seen only in ECFCs treated with mango. Finally, a general activation of ECFC angiogenic capacity was seen after 72 h of incubation with all the extracts, presenting a higher number of meshes, segments, and longer segments than control ECFCs. Nevertheless, mango was the extract that stimulated the highest pro-angiogenic affect, even greater than the one exerted by the angiogenic activator (*p*-values < 0.001 in ECFCs + Mango vs ECFCs + FGF).

### 3.4. Olive, Grape and Mango Extracts Decrease the Proliferation of ECFCs and Promote Their Differentiation into Mature ECs

**Figure 3:**
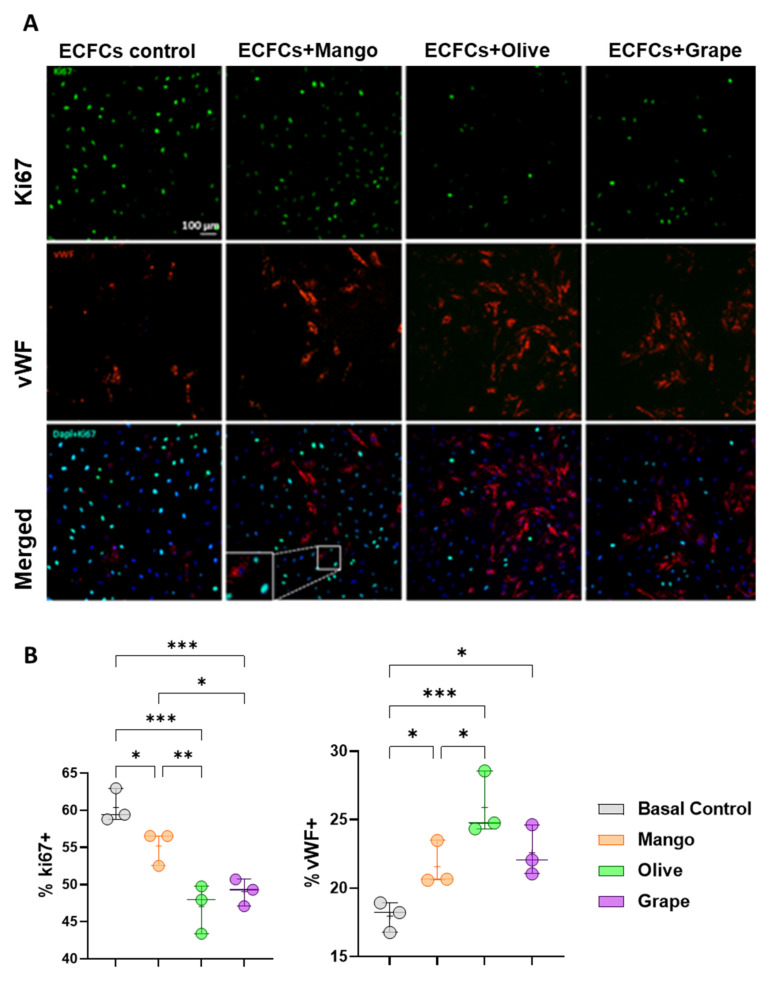
Effect of the selected extracts on ECFC proliferation and differentiation. (**A**) Representative images of Ki67 and vWF staining of ECFCs treated 48 h with the ethanolic extracts of mango (4.99 μg/mL) and olive (46.67 μg/mL) leaves, and red grape pomace (11.67 μg/mL). (**B**) Box and whisker diagrams of the effect of the selected extracts on the proliferation and differentiation of ECFCs after 48 h of treatment. * *p*-value: * *p* < 0.05; ** *p* < 0.01; *** *p* < 0.001.

All three extracts, mango, olive, and grape, led to a significant decrease in proliferation and higher differentiation levels in ECFCs incubated 48 h with any of the extracts, compared to basal ECFCs. Overall, a greater reduction in proliferation was related to a greater increase in differentiation. Among the extracts, the decrease in ECFC proliferation was less pronounced with mango, compared to olive or grape extracts, where a 10% decreased proliferation was seen vs control cells (Figure 3). Thus, olive was the extract that promoted greater differentiation of ECFCs at the expense of lower proliferation, followed by grape and mango. In fact, significant differences were observed when comparing the effect of mango and olive, since olive promoted notably higher anti-proliferation and pro-differentiation effects than mango.

### 3.5. Extracts Reduce Apoptosis in ECFCs

**Figure 4:**
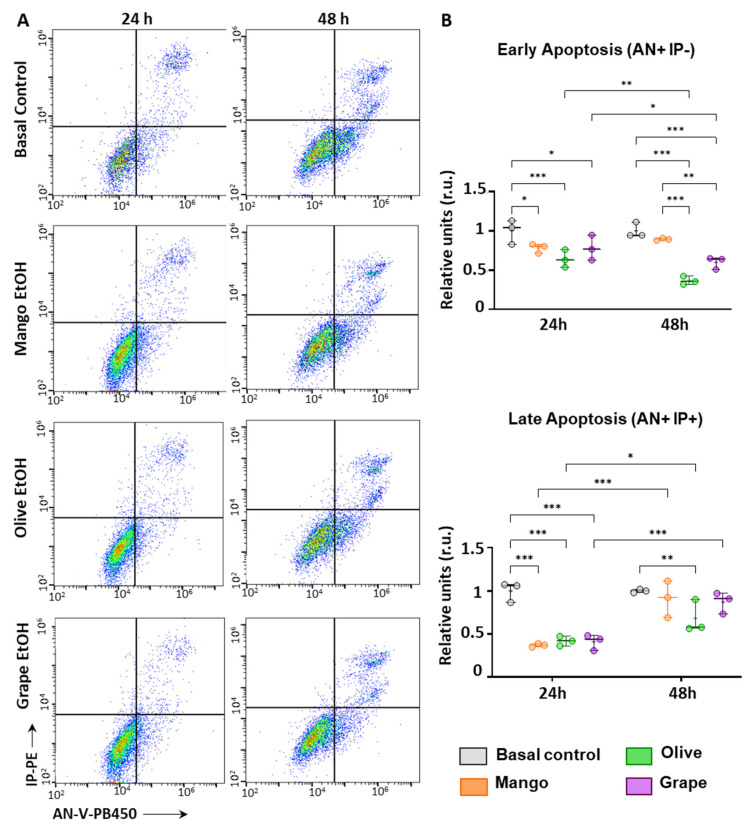
Effect of the selected extracts on the apoptosis of ECFCs. (**A**) Representative dot-plots of mango leaves, olive leaves, and red grape pomace extracts effect on ECFCs apoptosis after 24 and 48 h of treatment. (**B**) Graphical representation of the effect of mango leaves (4.99 μg/mL), olive leaves (46.67 μg/mL), and red grape pomace (11.67 μg/mL) on early and late apoptosis in ECFCs after 24 and 48 h of treatment. * *p*-value: * *p* < 0.05; ** *p* < 0.01; *** *p* < 0.001.

The incubation of ECFCs for 24 and 48 h with all three extracts promoted a reduction in the number of pre-apoptotic cells (AN-V+, IP-), compared to basal controls. This anti-apoptotic effect was more noticeable after 48 h of treatment with grape and olive extracts, while the down-regulation in the number of pre-apoptotic cells was lower in ECFCs treated with mango (Figure 4).

Similarly, the three extracts strongly inhibited the number of late apoptotic cells (AN+/IP+) after 24 h of treatment. On the other hand, after 48 h, although the number of apoptotic cells was still lower in ECFCs treated with olive, mango, or grape, compared to ECFCs control, the highest anti-apoptotic effect was seen with the olive leaf extract (** *p*-value < 0.01).

### 3.6. Extracts Exert an Anti-Inflammatory Effect in ECFCs

**Figure 5:**
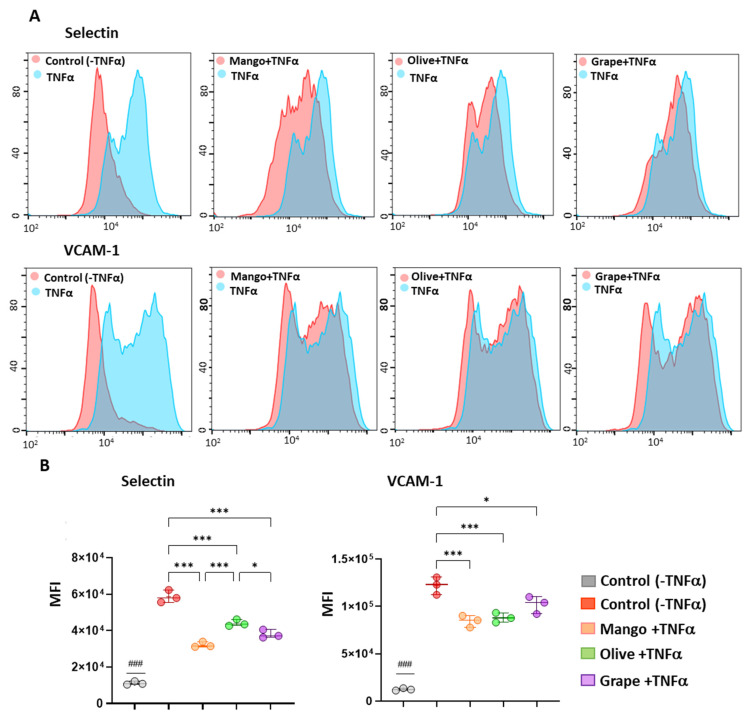
Anti-inflammatory effect of selected extracts. (**A**) Representative histograms of the effect of treatment with TNFα alone and TNFα with the mango (4.99 μg/mL), olive (46.67 μg/mL), and grape (16.67 μg/mL) ethanolic extracts on the expression of the adhesion molecules E-selectin and VCAM-1, at 4 h. (**B**) Box and whisker diagrams of the effect of selected extracts on the expression of adhesion molecules. MFI: Mean Fluorescence Signal. ### Significant differences with respect to the control condition (-TNFα). * Significant differences between the conditions. * *p* < 0.05; *** *p* < 0.001.

The three extracts exerted an anti-inflammatory effect in ECFCs, significantly decreasing the expression of both adhesion molecules compared to control cells with TNFα (Figure 5). Among the extracts, mango promoted the highest reduction of E-selectin levels compared to control cells and also compared to olive or grape. As for VCAM-1, mango was again the extract that decreased its expression the most, followed by grape and olive.

## 4. Discussion

Therapeutic angiogenesis represents a novel strategy that allows the reconstitution of the damaged vascular network in CVD patients. Due to their great angiogenic capabilities, ECFCs are the main candidates for vascular repair approaches; however, when these cells are exposed to inflammatory and oxidative environments, their regenerative role is adversely affected. Therefore, different studies have evaluated the potential of pre-stimulating ECFCs with antioxidant substances prior to cellular therapy in order to enhance their regenerative properties and reduce the negative effects of such pathological environments, finding promising results [13,16].

In the current study, we analyzed the effect of three extracts rich in polyphenols, mango leaves, olive leaves, and red grape pomace extracts, over ECFCs. Ethanolic extracts were chosen rather than aqueous ones since they presented higher antioxidant capacity, especially with mango leaves. Moreover, the ethanolic extracts also exerted a greater pro-angiogenic effect than aqueous, at least when using low concentrations. Previous studies have demonstrated that the use of antioxidant substances such as polyphenols, such as those previously determined in the extracts analyzed herein, correlates with an improvement of their angiogenic abilities [13].

Despite this, we also observed that higher ethanolic concentrations decreased the angiogenic capacity of ECFCs. The anti-angiogenic effect was especially notable when 140 µg/mL of the ethanolic olive leaves extract was used, in agreement with the results seen with BALB-c mice with breast tumour implants treated for three weeks with 225 mg/kg/day of olive tree leaf extract (from a mixture of aqueous acetate buffer and acetonitrile as solvent). Such treatment reduced tumour angiogenesis and stimulated apoptosis of tumoral cells [33]. Thus, based on these preliminary results, we focused on evaluating the effect of ethanolic mango leaves, olive leaves and red grape pomace extracts at concentrations of 4.99, 46.67 and 16.67 µg/mL, respectively, over ECFCs.

According to our results, mango was the extract with the strongest angiogenic potential, probably related to the major polyphenol content and antioxidant activity. Similarly, previous studies demonstrated that mango leaves extract promotes ECs migration, favoring the angiogenic process, although these effects were dependant on the isolated polyphenols present in the extract. Thus, while mangiferin promoted migration, quercetin inhibited it. Therefore, the balance between different components might be decisive in the effect exerted by the extract [34]. Future studies should determine the effect of individual mango extracts components over ECFCs.

Grape extract, rich in anthocyanins and phenolic acids, also exerted a pro-angiogenic effect over ECFCs, although to a lesser extent than mango. A study analysing the angiogenic properties of anthocyanins and fatty acids from blueberry extracts (similar to the grape extract) reported that anthocyanins inhibited angiogenesis, while the isolated phenolic acids as well as the combined treatment with anthocyanins and phenolic acids promoted it [35]. Additionally, resveratrol, a polyphenol contained in red grape skin, was found to enhance angiogenesis on umbilical cord vein endothelial cells (HUVECs) [36], supporting the results observed in the present study.

Finally, olive extract also improved the angiogenic process in ECFCs, only 72 h after treatment. Similarly, previous results revealed that low concentrations (1–5 µM) of 3-hydroxytyrosol, one component of olive extract, stimulated migration and angiogenesis of ECs in vitro [37].

Despite the above, most researchers have reported an anti-angiogenic role for these extracts, mainly in cancer-related studies. For instance, mangiferin seems promising in the treatment of melanoma due to its anti-angiogenic and antimetastatic effects [38]. Similarly, oleuropein administration decreased angiogenesis and lymphangiogenesis in HUVECs and lymphatic endothelial cells (LECs), as well as prevented tumor progression in a murine melanoma model [39]. In addition, grape seed extracts (with polyphenolic composition similar to red grape pomace extract) exerted an anticancer effect in a murine model of prostate cancer, due to its anti-proliferative, pro-apoptotic and anti-angiogenic activity [40]. Therefore, although the conditions that lead the extracts to exhibit a pro- or anti-angiogenic behaviour remain unclear, this could be explained by either the cell type, or by the concentration of the extracts, as shown here.

Regarding proliferation, all three extracts showed anti-proliferative and differentiation-enhancing effects over ECFCs. These effects were stronger in response to the olive extract, followed by the pomace and mango. The anti-proliferative activity of the extracts can be supported by a wide range of studies. For instance, extracts from mango leaves or from other parts of this plant are capable of exerting a protective effect against different types of breast cancer, through its cytotoxic and anti-proliferative effect, which cannot be associated with a single component of the extract, but to the synergistic effect of the different polyphenols present [30,41]. Furthermore, grape pomace and olive leaf extracts seem to inhibit the proliferation of colon cancer cells [42] and different human carcinoma cell lines [43], respectively.

Regarding the increase in differentiation, olive leaves extract also promoted the differentiation of human mesenchymal stem cells (hMSCs) towards ECs, increasing the expression of vascular growth factor endothelial (VEGF), platelet-derived growth factor receptor (PDGFR) and the endothelial growth factor receptor (VEGFR-1) [44]. Furthermore, resveratrol, a polyphenol present in grape skin, induces the differentiation of vascular progenitor cells to endothelial cells [45].

The increase in differentiation as well as the consequent decrease in proliferation are related to the pro-angiogenic capacity of the extracts, given that the differentiation towards more mature ECs promotes the formation of tubular structures, an essential process in angiogenesis and vasculogenesis [44], as well as mobilization towards the injured area and vascular repair [46].

The three extracts also decreased apoptosis in ECFCs, more significantly with olive extract, followed by pomace and mango. Several studies support this anti-apoptotic effect, which may be associated with its antioxidant and anti-inflammatory capacities, reflecting a protective role [47,48,49]. The reduction of the apoptotic process would also support differentiation outcomes at the expense of a decrease in proliferating cells.

Finally, as already mentioned, the three extracts exhibited an anti-inflammatory activity on ECFCs, reducing the expression of endothelial adhesion molecules VCAM-1 and E-selectin. The anti-inflammatory effect of the analyzed extracts has been previously reported [50,51,52] and, along with the antioxidant activity, may also be related to a protective effect on ECFCs.

Of note, vWF up-regulation is often associated with endothelial damage and inflammation, platelet aggregation, and adhesion, and it is considered as a biomarker of atherosclerosis and thrombogenesis, among others [53]. In the current study, however, considering that the extracts reduced the inflammatory response in ECFCs, the increased expression of vWF should not be associated with endothelial dysfunction, but with greater maturation of these cells [53].

## 5. Conclusions

Overall, our findings suggest that low concentrations of mango leaf, olive leaf and red grape pomace ethanolic extracts can exert a pro-angiogenic, anti-proliferative, and anti-apoptotic effect on ECFCs. Moreover, these extracts reduced the inflammatory response of these cells and promoted ECFCs differentiation into more mature cells. It is worth noting the great pro-angiogenic power of the mango leaves extract, which was much higher than that exhibited by the other two extracts studied. These effects are closely related to its antioxidative properties. Further studies are needed to determine whether the pre-treatment of ECFCs with these extracts could boost cell therapy and revascularization in vivo.

## Data Availability

Data is contained within the article and Supplementary Material.

## Supplementary Materials

The following supporting information can be downloaded at: https://www.mdpi.com/article/10.3390/antiox11050851/s1. Figure S1: Viability tests; Figure S2: Preliminary angiogenesis test; Figure S3: Characterization of ECFCs. Table S1: Antibodies used for cytometry analyses and immunofluorescence.
