# Supplementary material for: Pro-Angiogenic Effects of Natural Antioxidants Extracted from Mango Leaf, Olive Leaf and Red Grape Pomace over Endothelial Colony-Forming Cells
Source: Antioxidants (Basel). 2022 Apr 27;11(5):851. doi: 10.3390/antiox11050851 (PMC9137485; doi:10.3390/antiox11050851)
Supplement: Supplementary file 1 [file antioxidants-11-00851-s001.zip › antioxidants-1703205-supplementary.pdf]

Supplementary Table S1: Antibodies used for cytometry analyses and immunofluorescence. FC: Flow cytometry.

Antibody	Species	Dilution	Supplier	Reference	Used in
**Primary antibodies**					
CD14-PB	Human	1:25	Biolegend	#367121	FC
CD31-FITC	Human	1:25	Biolegend	#303103	FC
CD34-APC	Human	1:25	Biolegend	#343607	FC
CD45-PB	Human	1:25	Biolegend	#368539	FC
CD73-FITC	Human	1:25	Biolegend	#303103	FC
CD90-APC	Human	1:25	Biolegend	#328109	FC
CD133-PE	Human	1:25	Miltenyi Biotec	130-098-826	FC
CD146-PE	Human	1:25	Biolegend	#361005	FC
CD309-PE	Human	1:25	Biolegend	#359903	FC
IgG1 isotype	Human	1:25	Becton-Dickinson	345816	FC
-Ki67 antibody	Human, rat	1:500	Invitrogen	PA5-16785	IF
VWF	Human	1:200	Thermo Fisher Scientific	MA5-14029	IF
**Secondary antibodies**					
Alexa Fluor 488	Rabbit	1:1000	Thermo Fisher Scientific	A-11008	IF
Alexa Fluor 555	Rabbit	1:500	Thermo Fisher Scientific	A-21422	IF

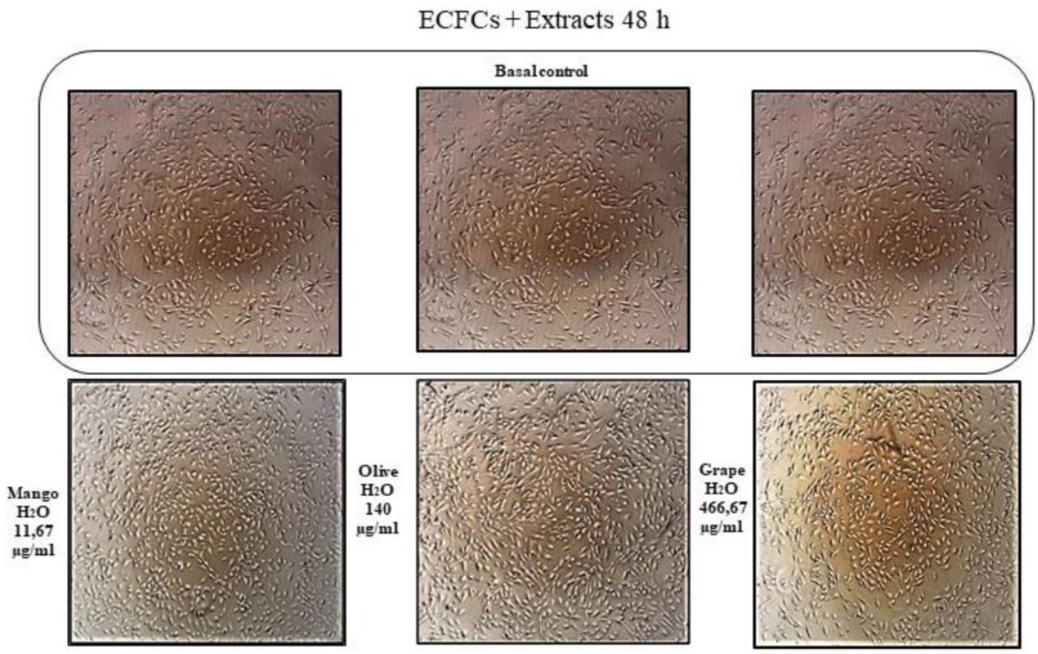

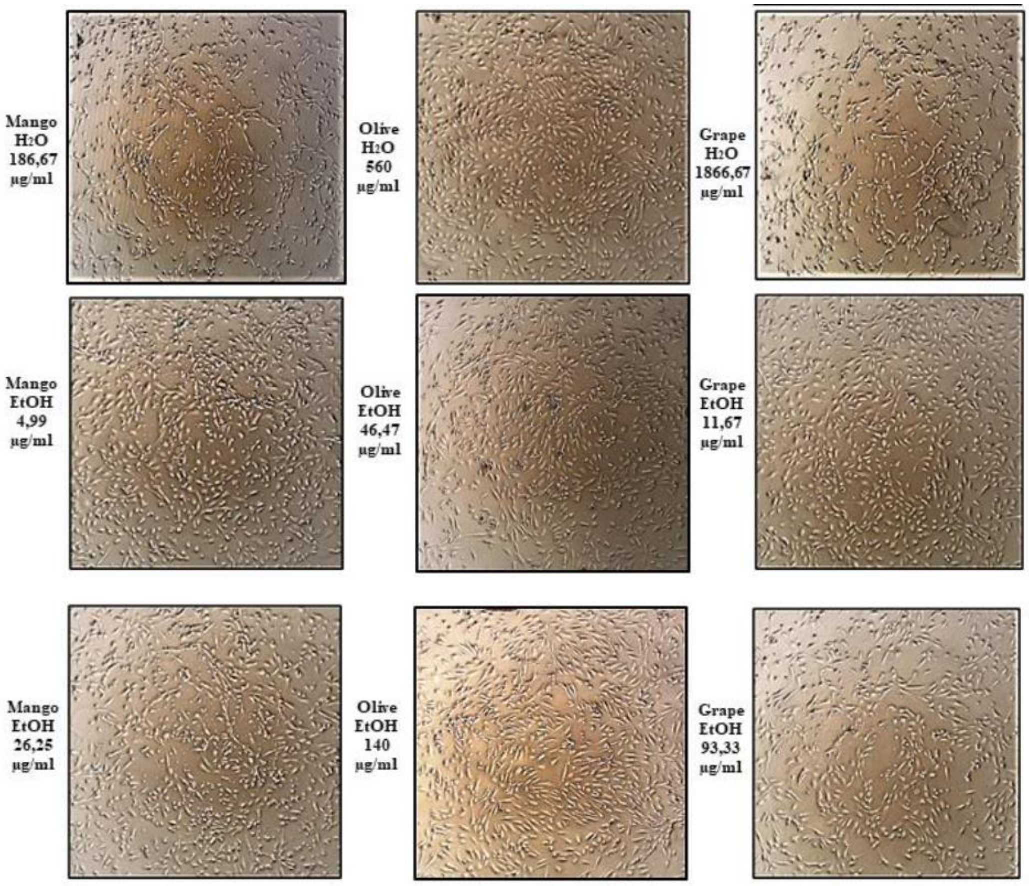

Supplementary Figure S1: Viability test. Images taken for ECFCs after 48h of culture with different concentrations of the extracts. The rank of concentrations that kept high cell viability was 11.67-186.67 and 4.99-26.25 µg/ml for the aqueous and ethanolic mango leaves extracts respectively, 140-560 and 46.67-140 µg/ml in the aqueous and ethanolic olive leaves extracts, and finally 466,67-1866,67 and 11.67-93.33 µg/ml in the aqueous and ethanolic red grape pomace extracts.

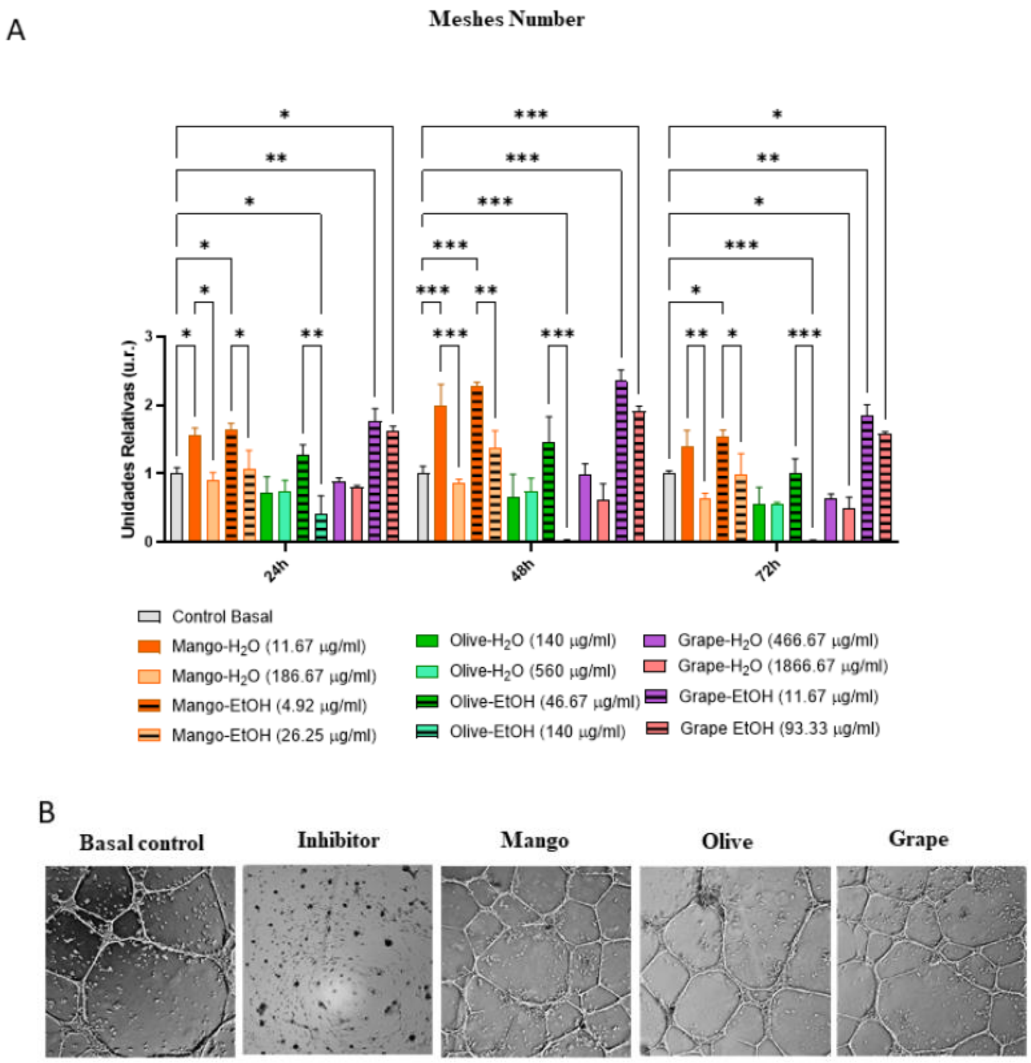

Supplementary Figure S2. Preliminary angiogenesis test. A) Graphical representation of the number of meshes after 24, 48 and 72h after the treatment with high and low concentrations of the aqueous and ethanolic extracts. The lower concentrations of the ethanolic extracts provided the major number of meshes. **B**) Representative images of the reticular structures formed by ECFCs after the treatment with the ethanolics extracts of mango leaves (4.99 µg/ml), olive leaves (46.67 µg/ml) and red grape pomace (11.67 µg/ml), compared with the basal control and treatment with inhibitor (15 Mm). *$p < 0.05$; **$p < 0.01$; ***$p < 0.001$

Once the range of concentrations that kept the viability of the culture was stablished, two concentrations per extract were selected, corresponding to the two extremes of the intervals, the lowest and highest concentrations that maintained the viability of the culture. An angiogenesis test was performed by subjecting the extracts to said concentrations to determine which extracts, aqueous or ethanolic, and what concentrations improve the angiogenic capacities of the ECFCs to a greater extent.

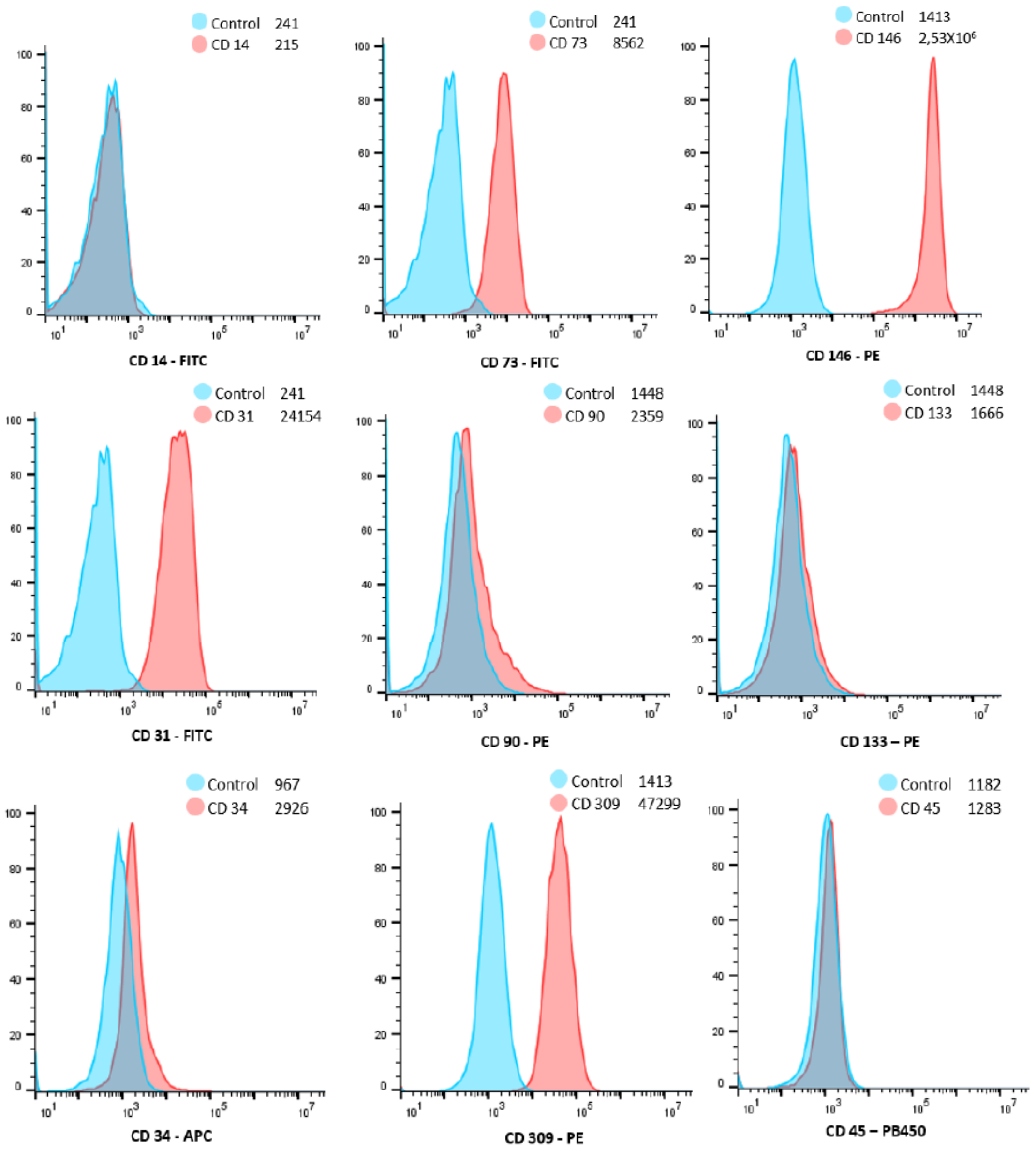

Supplementary Figure S3: Characterization of ECFCs. Cell identity was confirmed by flow cytometry, analyzing several specific antibodies against CD31, CD14, CD90, CD34, CD45, CD73, CD133, CD309 and CD146. An isotype IgG1 antibody was used for negative control. Data were presented as Mean Fluorescence Intensity (MIF) normalized.
